# Photoassisted Batch Injection Analysis (photoBIA): Monitoring Photoelectrochemical Activity of Catalysts

**DOI:** 10.1021/acsomega.5c09379

**Published:** 2025-11-10

**Authors:** Luiz Eduardo Gomes, Vitória E. M. Icassatti, Andréa L. C. D. de Avelar, Zanib Qazi, Rodrigo A. B. da Silva, Heberton Wender, Cauê A. Martins

**Affiliations:** a Institute of Physics, 54534 Universidade Federal de Mato Grosso do Sul (UFMS), CP 549, Campo Grande, Minas Gerais 79070-900, Brazil; b Institute of Chemistry, Universidade Federal de Uberlândia (UFU), Uberlândia, Minas Gerais 38400-902, Brazil

## Abstract

We introduce photoassisted batch injection analysis (photoBIA), a novel analytical system that integrates photoelectrochemistry with batch injection analysis (BIA), as a sensitive, reproducible, and time-resolved method for evaluating the activity of light-sensitive semiconductor electrodes. Using BiVO_4_ and α-Fe_2_O_3_/TiO_2_ as model semiconductor photoanodes and alcohols (methanol and glycerol) as probe molecules, the photoBIA enables precise delivery of analytes onto an illuminated photoelectrode through hydrodynamic injection, generating sharp faradaic current transients, characteristic of photoelectrooxidation events. The system was benchmarked against conventional stationary photoelectrochemical cells, which exhibited a limited mass transport and low temporal resolution. In contrast, photoBIA allowed for rapid detection of interfacial processes with clear discrimination under light and dark conditions. These results demonstrate the potential of photoBIA for rapid screening of photosensitive catalysts under well-controlled and realistic conditions, providing information for evaluating potential photoanodes or photocathodes in fuel cell development and light-driven electrochemical systems.

## Graphical abstract

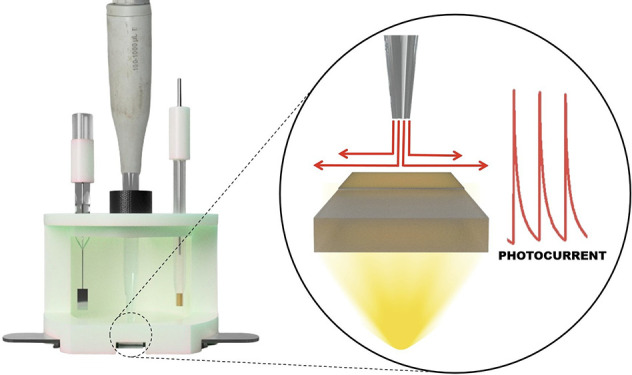

## Introduction

Photosensitive materials, such as semiconducting metal oxides, have garnered growing interest as key components in photoelectrochemical fuel cells and electrolyzers due to their ability to harvest solar energy and drive redox reactions with enhanced selectivity and energy efficiency.^1,2^ By generating electron–hole pairs under illumination, these materials enable light-assisted oxidation and reduction processes, offering sustainable alternatives for energy conversion and environmental remediation. Materials like BiVO_4_,^3^ WO_3_,^4^ and black-TiO_2_ ^2^ have shown promising performance in alcohol oxidation and water splitting under simulated sunlight, opening new pathways for low-energy and solar-driven technologies.

Despite their potential, advancing the application of photosensitive materials requires the identification of catalysts that are not only active under illumination but also stable and selective under operational conditions. A central challenge in this pursuit is the accurate and reproducible characterization of photoelectrochemical activity.^5^ Researchers often use stationary electrochemical cells with diffusion-driven mass transport, but these systems limit current signals (intensity and precision) and fail to reflect real scenarios where convection dominates. The sluggish and nonuniform transport of reactants to the electrode surface can obscure the detection of transient phenomena and reduce the visibility of subtle photoresponses, which are critical especially in the presence of a low analyte concentration or near-threshold illumination intensities.

In this context, batch injection analysis with amperometric detection (BIA-AD) presents a compelling alternative.^6^ Usually, BIA-AD is a hydrodynamic electrochemical system in which a small sample volume (mL order) is rapidly injected onto the working electrode surface submerged in a larger volume of supporting electrolyte (mL order). By using electronic pipettes, the injection creates a controlled flow of analyte toward the electrode, resulting in reproducible transient peak-shaped currents that correspond to the redox activity of electroactive species under a suitable applied potential.^7^ Unlike continuous flow systems (e.g., FIA, flow injection analysis), BIA does not require fixed pumps or carrier tubes (avoiding bubbles) and operates under discontinuous flow (only during the pipette injection). As a consequence, BIA may be more user-friendly, simple, quick, portable, and robust than FIA.

The wall-jet-like configuration of BIA enhances faradaic signal detection and makes it ideal for studying rapid redox reactions under well-defined hydrodynamic conditions. Originally developed and widely explored for quantitative electroanalysis,^6,8,9^ BIA-AD offers several advantages such as precise control of injection timing and volume, low reagent consumption, rapid signal acquisition, and improved signal-to-noise ratio due to convective mass transport.^6^ These attributes enable real-time monitoring of redox reactions at high reproducibility and temporal resolution.^10^

Recent studies have highlighted the versatility of the wall-jet configuration in electrocatalysis, encompassing applications from materials preparation^11,12^ to assessing the electrocatalytic performance of nanoparticle-modified electrodes in alcohol electrooxidation.^13,14^ These approaches revealed catalytic aspects not observable in traditional setups (stationary). However, to date, there are no reports of BIA being used to investigate photosensitive materials or to monitor photoelectrochemical reactions. The integration of BIA with light-activated systems remains unexplored despite its potential to enhance the temporal and spatial resolution of photocatalytic activity measurements.

Here, we introduce photoassisted batch injection analysis (photoBIA), a hybrid system for evaluating the photoelectrochemical activity of semiconducting electrodes. Using BiVO_4_ and α-Fe_2_O_3_/TiO_2_ as model photoanodes, we demonstrate the system’s ability to clearly enlighten the photoelectrooxidation of methanol under simulated sunlight, also illustrated for glycerol photoeletrooxidation. The method enables rapid, reagent-efficient, and selective detection of photoinduced faradaic processes, overcoming limitations of diffusion-controlled systems and offering a powerful new tool for the screening of photoactive materials in fuel cell and electrolyzer research.

## Experimental Section

BiVO_4_ catalyst was synthesized as previously reported.^15^ Figure S1 illustrates the BiVO_4_ film synthesis on transparent fluorine-doped tin oxide (FTO)-covered glass substrates. Figure S2 shows the preparation of the α-Fe_2_O_3_/TiO_2_ film. Briefly, an iron (Fe) thin film was deposited onto a fluorine-doped tin oxide (FTO) substrate by DC magnetron sputtering under an argon flow rate of 50 sccm and a sputtering power of 50 W for 2 min. Subsequently, a thin titanium (Ti) layer was deposited on top of the Fe film under identical sputtering conditions for 10 s. The resulting sample was then annealed at 550 °C for 30 min in air to promote the crystallization of the Ti-modified hematite (α-Fe_2_O_3_/TiO_2_) structure. The photoactivities of BiVO_4_ and α-Fe_2_O_3_/TiO_2_ for methanol (and glycerol oxidation for BiVO_4_) were tested in a standard photoelectrochemical cell^3,16^ and in the proposed photoBIA cell for comparison. All measurements were conducted in a specific electrolyte containing either methanol or glycerol using a photoanode working electrode (WE), a platinum foil counter electrode (CE), and a Ag/AgCl reference electrode (RE) all connected to a CS350 M CorrTest potentiostat. Linear voltammograms were recorded at a scan rate of 10 mV s^–1^, and chronoamperometry was performed at potentials indicated in each case. The photoBIA cell was equipped with a Kasvi electronic single-channel micropipette, and the bottom part was specially adapted for accommodating a 2 × 1 cm photoanode with an illuminated active area of 0.32 cm^2^ (Figure 1). The photoanode was illuminated with a solar simulator equipped with a 150 W Xe lamp (model 105000, Abet Tech) coupled to an AM 1.5G filter operating at 100 mW cm^–2^, calibrated using a reference solar cell (model 15151, Abet Tech).

**Figure 1:**
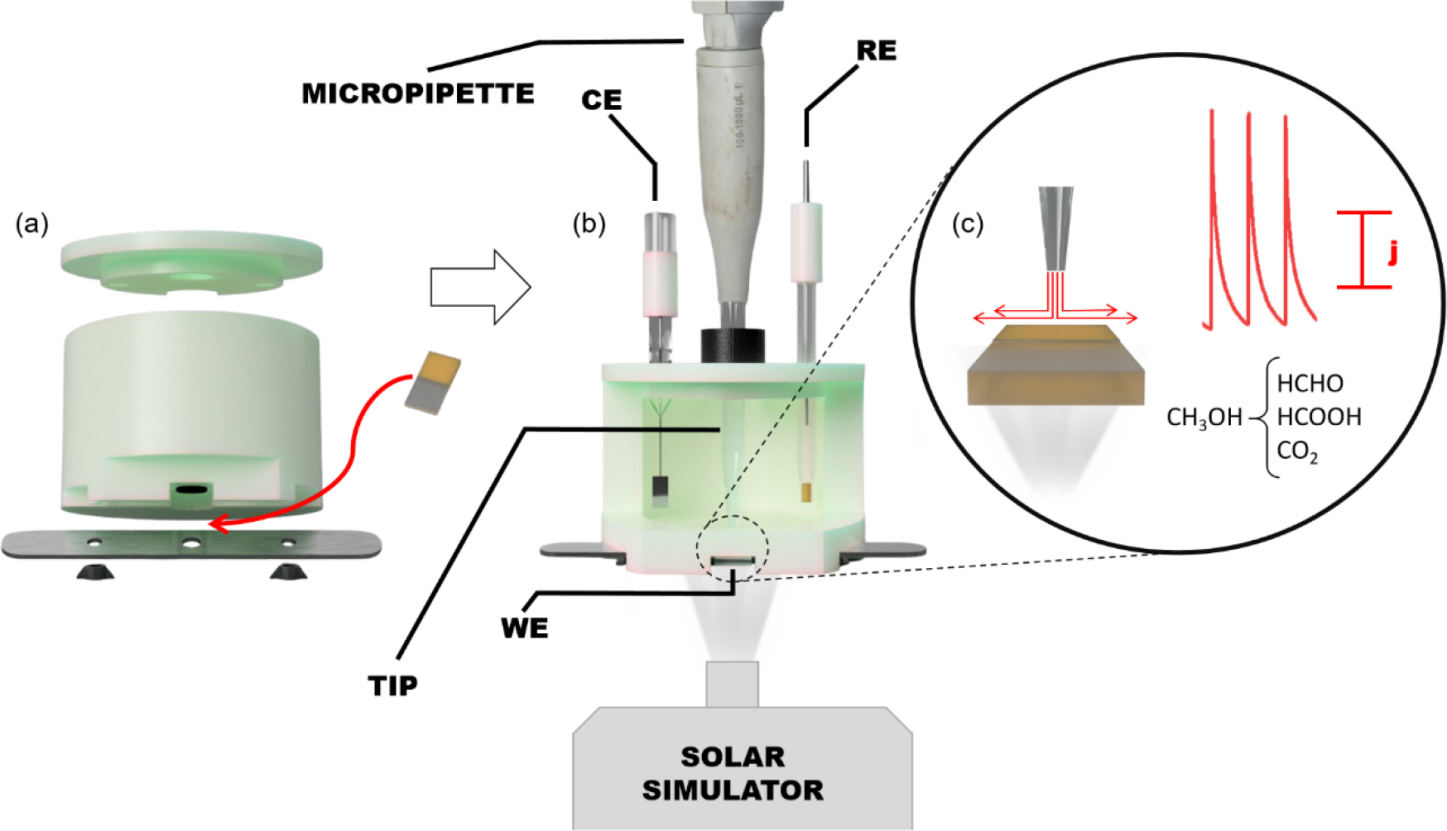
(a) Disassembled photoBIA cell indicating the placement of the WE. (b) Assembled photoBIA cell with an orthogonal cut highlighting the CE, RE, and WE, along with batch injection via electronic micropipette beneath a solar simulator. (c) Scheme of the dispersion of the oxidized species during the injection over WE and respective generated transient current responses for three injections. The products from methanol photoeletrooxidation are merely illustrative.

Figure 1 shows the design, assembly, and functioning of the photoBIA cell, which is adapted from an earlier system.^17^ Figure 1a illustrates the disassembled photoBIA cell to highlight the placement of the planar WE at the base of the cell in a recessed cavity. The modular assembly includes sealing components and support structures that ensure proper electrode alignment and leak-free operation. More details of the cell are shown in Figure S3. The aperture at the base permits light to pass through the FTO, thereby activating the photoanode for the surface reaction. Electrical contact is established directly on the FTO. Figure 1b shows the assembled photoBIA cell with an illustrative orthogonal cut, revealing its internal configuration. RE and CE are inserted vertically at the cover of the cell and positioned around the WE. Also, in the cover, an electronic micropipette assembled with a blue tip is positioned to deliver controlled injections of analyte solution (*v* = 100 μL) directly onto the WE surface. Below the base of the cell, a solar simulator provides continuous or pulsed illumination during measurement, activating the photosensitive material to initiate photoelectrochemical reactions. The vertical injection axis mimics a wall-jet configuration, where the injected volume reaches directly on the electrode surface before spreading radially, promoting convective mass transport.

Figure 1c schematically summarizes the working principles of the photoBIA system. Upon hydrodynamic injection of an analyte such as methanol (CH_3_OH) onto the illuminated photoelectrode, photogenerated holes in the valence band of the semiconductor oxidize the organic molecules.^2,18^ This may lead to the formation of products, such as formaldehyde (HCHO), formic acid (HCOOH), and CO_2_. These oxidation processes give rise to transient peak current signals, which reflect the passage of the liquid sample zone over the WE. Each peak in the current–time profile corresponds to a distinct injection event, enabling precise correlation between the electrochemical response and the presence of the target analyte.

## Results and Discussion

The technique was demonstrated through fast detection of the photoelectrochemical activity of BiVO_4_ photoanodes for alcohol oxidation. Figure 2 presents a comprehensive comparison between traditional photoelectrochemical characterization (Figure 2a) and the newly developed photoBIA system (Figure 2b) using BiVO_4_ as a model photoanode for the photoelectrooxidation of methanol. Figure 2a shows the linear sweep voltammetry profiles recorded in a conventional photoelectrochemical cell in the presence and absence of methanol. In the absence of light (dotted black curve), the BiVO_4_ photoanode exhibits negligible current density over the entire potential range, confirming its electrochemical inertness in the dark. Under simulated solar illumination in the presence of 3 mol L^–1^ KCl (red curve, pH 7.01), the photocurrent response begins to rise at approximately 0.05 V *vs*. Ag/AgCl, associated with the onset of the oxygen evolution reaction (OER) on the BiVO_4_ surface. When methanol (20% v/v) is introduced into the electrolyte (blue curve, pH 6.79), a significant enhancement in the photocurrent is observed, accompanied by a decrease in the onset potential to approximately −0.10 V *vs*. Ag/AgCl. This result highlights two key features of BiVO_4_ photoanodes: first, their capacity to oxidize the alcohol more efficiently than water and, second, their ability to exploit the kinetic advantage of methanol oxidation, resulting in higher photocurrent densities and earlier activation under illumination. Such behavior is consistent with the role of photogenerated holes in facilitating multielectron transfer reactions at the semiconductor–electrolyte interface.

**Figure 2:**
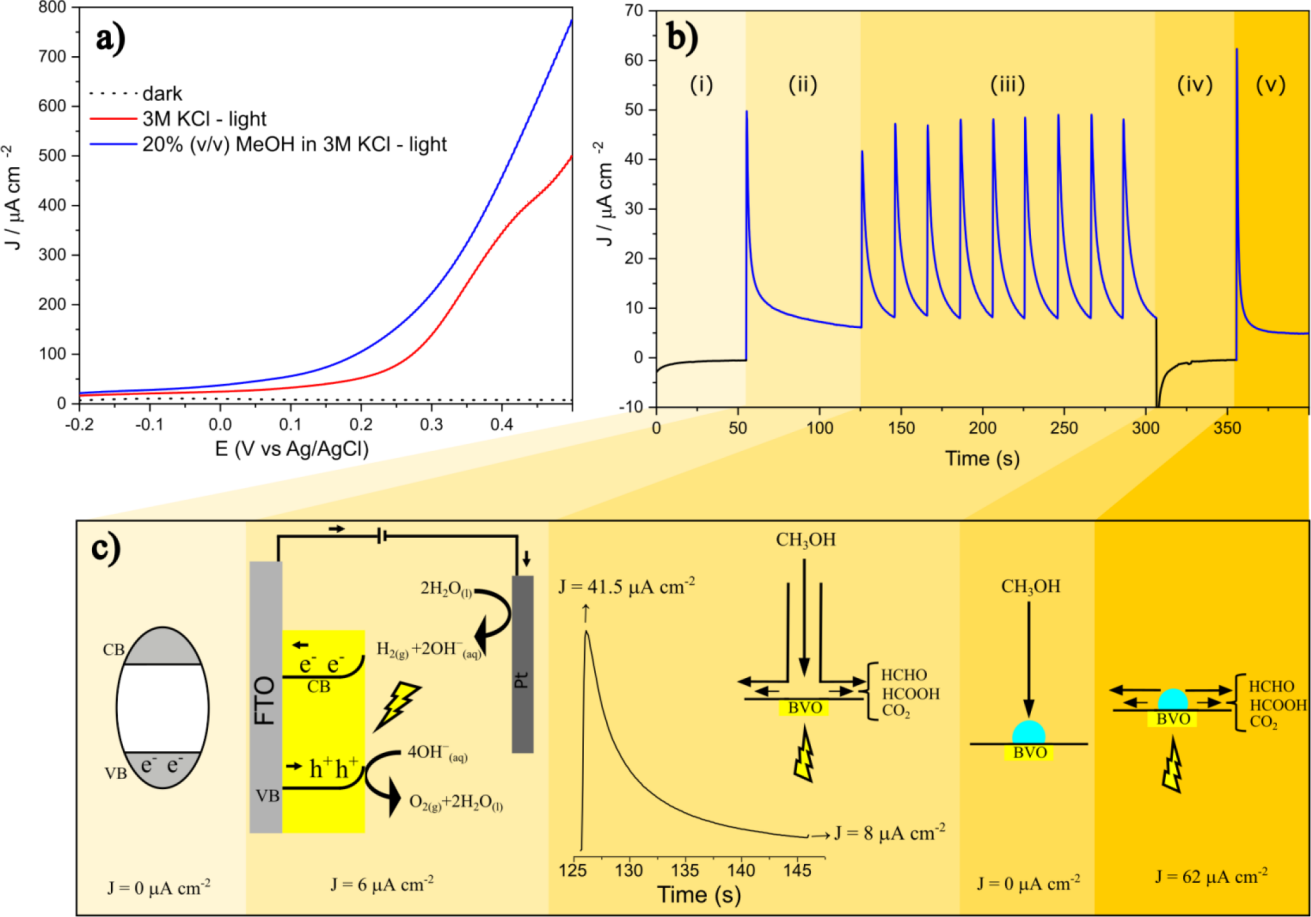
(a) Water and methanol photoelectrooxidation in a conventional stationary cell (scan rate = 10 mV s^–1^). (b) Transients of water and methanol photoelectrooxidation measured in a photoBIA system with amperometric detection (*E* = 0.1 V), highlighting five different regions: (i) dark condition with no photocurrent; (ii) light on, showcasing water oxidation only; (iii) sequential MeOH injections under illumination, producing photoinduced current peaks; (iv) MeOH injections in the dark showing no response; and (v) light turned back on, leading to oxidation of residual MeOH at the BiVO_4_ surface. Injections were electronically controlled using an electronic micropipette preconfigurated to inject 100 μL under dark or light conditions at 100 mW cm^–2^. (c) Schematic explanation of the different events, where MeOH oxidation products are shown for illustration only.

Figure 2b displays the chronoamperometric response of BiVO_4_ measured under injections of methanol into the photoBIA system. The current–time curve is segmented into five distinct regions, each corresponding to a specific experimental condition. In region (i), the cell is held in the dark without any injection (reflecting a stationary cell), and no current is detected, as expected for an unilluminated semiconductor. Upon illumination, in region (ii), an abrupt increase in the photocurrent appears, followed by a continuous decay, reflecting insufficient mass transfer during water photooxidation. After some time (≈75 s), the system achieved a photocurrent baseline of approximately 6 μA cm^–2^, reflecting slow water oxidation. This background serves as a reference for subsequent analyte-induced responses.

Region (iii) in Figure 2b showcases the defining advantage of the photoBIA configuration. Here, nine repeated injections of methanol (100 μL each at intervals of 20 s between injections) are delivered onto the illuminated BiVO_4_ surface. Each injection produces a rapid and intense current spike, with peak current densities exceeding 40 μA cm^–2^. These transient responses are characteristic of a convective mass transport regime and are indicative of the immediate photoelectrooxidation of methanol at the semiconductor interface. The temporal resolution afforded by the injection strategy enables the direct visualization of analyte arrival, surface interaction, and depletion dynamics. The exponential decay following each peak reflects the dilution and radial dispersion of the analyte as it moves away from the electrode surface, effectively modeling a single-pass reaction zone. This type of transient signal is not only a fingerprint of the electrochemical activity but also a powerful diagnostic of the photoanode efficiency under operationally relevant conditions.

In region (iv), the light source is turned off, and three additional methanol injections are performed in the dark. Notably, these injections produce no detectable photocurrent but noise signal, reinforcing the strict dependence of methanol oxidation on the presence of photogenerated charge carriers. Even at high local methanol concentrations, the absence of valence band holes under dark conditions renders the BiVO_4_ surface catalytically inactive, confirming that the observed faradaic processes are governed mostly by photoactivation. Finally, region (v) provides further insight into the surface and interfacial phenomena. Immediately following the reintroduction of light, a pronounced anodic current is observed, even without new methanol injections. This response is attributed to the oxidation of methanol molecules that had accumulated or adsorbed at the electrode surface during the preceding dark phase, allied to water oxidation. The capacity of the system to oxidize residual, nondiffusing analyte upon illumination not only demonstrates the sensitivity of photoBIA to interfacial species but also suggests its utility in probing adsorption–desorption kinetics and surface fouling processes.

Figure 2c offers a schematic summary of the photoelectrochemical events corresponding to each experimental region. In the dark (i), the valence band (VB) electrons of BiVO_4_ remain unexcited, preventing charge separation and current flow. Upon illumination (ii), excitation of electrons to the conduction band (CB) leaves holes in the VB and induces downward band bending at the BiVO_4_ photoanode surface, which facilitates charge separation and enables water oxidation (4OH^–^_(aq)_ → O_2(g)_ + 2H_2_O_(l)_ + 4e^–^), establishing a photocurrent. The excited electrons flow through the external circuit to the counter electrode, where water reduction occurs (2 H_2_O_(l)_ + 2e^–^→ H_2(g)_ + 2 OH^–^_(aq)_). During methanol injection under light (iii), the photogenerated holes drive the oxidative conversion of CH_3_OH into partially and fully oxidized products such as HCHO, HCOOH, and CO_2_. The resulting current peaks correspond directly to analyte oxidation events. With the light off (iv), no photogenerated carriers are available, and the reaction ceases, irrespective of the methanol concentration. Upon reillumination (v), rapid oxidation of previously adsorbed methanol and water yields a sharp transient current, reaffirming the system’s reactivity and temporal sensitivity.

Additional experiments were conducted by stirring the solution while injecting methanol onto BiVO_4_ using the photoBIA configuration, as well as in a different electrolyte commonly employed for BiVO_4_. Figure S4 shows voltammetric profiles under illumination, demonstrating a significant increase in photocurrent compared to the previous electrolyte due to more favorable operation conditions for BiVO_4_ (Figure S4a). Upon addition of methanol, the photocurrent increased by nearly a factor of 4 at 0.2 V *vs*. Ag/AgCl, indicating its favorable oxidation kinetics compared to water in borate buffer solution (BBS, pH 9.3). Without stirring, the injection of methanol produces distinct but relatively damped transient peaks (Figure S4b) due to the slow washing out of the analyte from the electrode surface by diffusion after injection. In contrast, introducing mechanical stirring amplifies these peaks, promoting more efficient delivery of methanol to the photoactive surface and enhancing the faradaic response (Figure S4c). This demonstrates the combined effect of photoactivation and increased mass transport, indicating the potential of the photoBIA platform for further analytical applications. In control tests, blank BBS was injected under illumination without stirring (Figure S4d) to verify that the current transients seen in Figure 2b,c are caused by methanol oxidation. This shows that the sharp current spikes in Figure 2b,c are linked to methanol oxidation.

**Figure 3:**
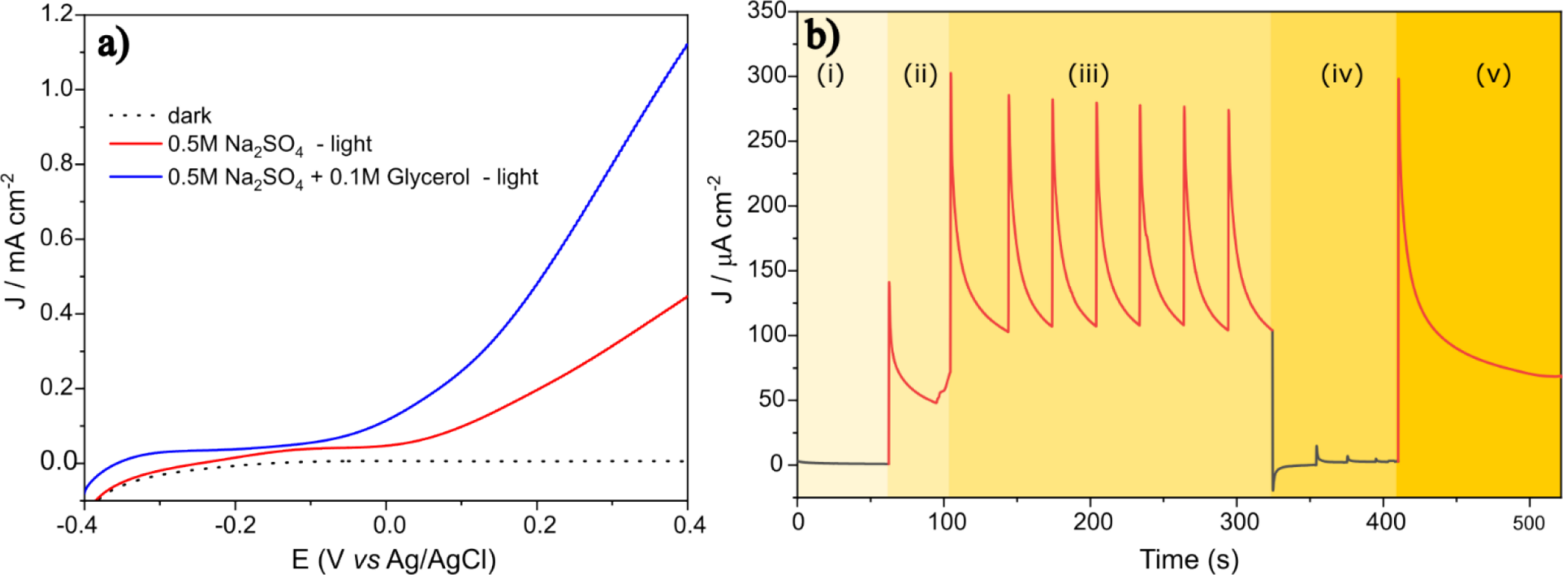
(a) Water and glycerol photoelectrooxidation in a conventional cell at 10 mV s^–1^. (b) Transients of water and glycerol photoelectrooxidation measured in a photoBIA cell. The presence of glycerol enhances photocurrent and shifts the onset potential to more negative values compared to water oxidation alone. (i) Dark condition with no photocurrent; (ii) light on with background water oxidation; (iii) sequential glycerol injections under illumination, producing sharp photoinduced current peaks; (iv) glycerol injections in the dark showing no response; and (v) light turned back on, leading to oxidation of residual glycerol at the BiVO_4_ surface. 100 μL was injected manually using an electronic micropipette under dark and light conditions at 100 mW cm^–2^.

Figure 3 further demonstrates the analytical power of the photoBIA platform by extending its application to the photoelectrooxidation of glycerol, a more complex and industrially relevant alcohol.^19^ In this set of experiments, BiVO_4_ was again used as the photoanode to evaluate both the baseline photoactivity in the presence of water and the catalytic response to glycerol under illumination. As observed previously with methanol, BiVO_4_ exhibited negligible electrochemical activity in the dark, confirming the absence of faradaic processes without photoexcitation in a conventional cell (Figure 3a). Under illumination in 0.5 mol L^–1^ Na_2_SO_4_ (red curve, pH 9.6), a photocurrent onset occurred near −0.10 V *vs*. Ag/AgCl, which is associated with the oxidation of water. When 0.1 mol L^–1^ glycerol was introduced (blue curve, pH 9.4), the photocurrent increased significantly across the entire potential range, and the onset potential shifted slightly to more negative values. This enhancement is indicative of the thermodynamically and kinetically more favorable oxidation of glycerol relative to water.

Figure 3b presents the time-resolved current response of the same system when operated in the photoBIA configuration. The experiment follows the same five-region structure previously described for methanol, offering a direct comparison of analyte features under identical measurement protocols. In region (i), the cell is kept in the dark without any glycerol injection, yielding no measurable current and confirming the necessity of light for catalyst activation. Upon illumination in region (ii), a small but stable background current appears, corresponding to the photoinduced oxidation of water in the absence of glycerol. In region (iii), glycerol is injected sequentially (*n* = 7), while the cell is continuously illuminated. Each injection produces a rapid current spike, with peak values exceeding 300 μA cm^–2^, significantly higher than those observed during methanol oxidation under comparable conditions. This increase is attributed not only to the higher number of oxidizable hydroxyl groups in glycerol but also to a potentially greater affinity of glycerol for the BiVO_4_ surface, promoting faster interfacial electron transfer.^20,21^ The transient current responses are highly reproducible and follow a rapid rise-and-decay profile characteristic of BIA systems operating under forced convective mass transport.

In region (iv) of Figure 3b, the light source is turned off, and additional glycerol injections are made. Differently from methanol, low anodic transients are seen, suggesting that glycerol and/or its partially oxidized compounds may be electrooxidized on BiVO_4_, which is easily identified in the BIA configuration. The current is very low compared to that under light, demonstrating once again that photoelectrooxidation on BiVO_4_ is highly dependent on photon absorption and the subsequent generation of charge carriers. Finally, in region (v), the light source is reactivated without further glycerol addition. A sharp anodic current spike is immediately detected, signaling the rapid oxidation of residual glycerol that had remained adsorbed (or nearby) on the BiVO_4_ surface during the dark phase.

In addition to the results described above, we also utilized the photoBIA cell to investigate methanol electrooxidation using an α-Fe_2_O_3_/TiO_2_ photoanode. Figure S5 shows a similar interpretation, proving the system regardless of the catalyst. The α-Fe_2_O_3_/TiO_2_ heterostructure exhibits a clear photocurrent response under alkaline conditions, enhanced by the presence of methanol. In the conventional cell (Figure S5a), methanol oxidation lowers the onset potential and increases the photocurrent density, consistent with facilitated hole transfer. In the photoBIA configuration (Figure S5a), sequential injections of methanol yield well-defined transients whose amplitudes grow proportionally with illumination intensity, from 100 to 200 mW cm^–2^, demonstrating the method’s sensitivity to photon flux.

These findings collectively highlight the versatility and robustness of the photoBIA platform as a simple diagnostic tool for the fast screening of photoelectrochemical reactions. The system’s high sensitivity to illumination-dependent faradaic events, ability to resolve rapid transient signals, and reproducibility affirm its analytical depth. Notably, the clear differentiation between analyte responses under light and dark conditions underscores the precise photochemical control that can be achieved with this method. By enabling real-time, low-volume, and convectively enhanced analysis of organic molecule oxidation, including both simple substrates such as methanol and structurally complex ones such as glycerol, photoBIA emerges as a practical and scalable strategy for probing material performance. Its inherent compatibility with miniaturization, automated injection, and a variety of illumination regimes opens a new avenue for the standardized screening of photoanodes and photocathodes. The strategy presented here could be adapted to the FIA if needed. Comparable outcomes can be achieved when the FIA operates in a discontinuous mode using successive injections. Nevertheless, photoBIA remains a good option due to its lower cost and its ability to prevent issues such as backflow and bubble formation, offering a more streamlined and reliable analytical process.

Beyond liquid-phase reactions, the intrinsic control and sensitivity of the photoBIA approach can be extended to the evaluation of gas-phase transformations, enabling accurate determination of the true faradaic currents associated with photoelectrochemical oxidation of molecules, such as CH_4_, and the reduction of CO_2_ under well-defined mass transport conditions. By integrating controlled gas delivery with convective hydrodynamic injections, the platform can minimize diffusion limitations and decouple photochemical contributions from parallel processes, offering a unique pathway to study kinetically challenging reactions relevant to solar fuel production and environmental remediation. This adaptability positions photoBIA as a diagnostic tool for aqueous-phase oxidation, and also a powerful method for probing light-driven activation of small molecules in liquid and potentially gas phases.

## Conclusions

This study presents the development and validation of photoBIA, an innovative technique system that merges the spatiotemporal control of batch injection analysis with the selectivity and sensitivity of photoelectrochemical activation. The use of BiVO_4_ as a photoanode under simulated sunlight enabled clear visualization of the photoelectrooxidation of methanol and glycerol through distinct, reproducible current transients. Importantly, the system proved to be capable of identifying kinetic and interfacial features often obscured in diffusion-limited setups. The photoBIA platform does not replace but rather complements conventional stationary measurements, offering enhanced mass transport through convective injections. A potentiostatic *I*–*t* measurement in a conventional stationary cell would primarily exhibit a monotonic current decay governed by diffusion, lacking the well-defined transient peaks observed under convective conditions in photoBIA, thereby adding valuable information to those obtained from traditional photoelectrochemical characterization. The technique’s adaptability to various operational modes (e.g., under stirring), compatibility with low-volume injections, and sensitivity to photon flux reinforce its potential as a robust analytical tool. photoBIA thus opens new possibilities for fast and reliable screening of photoactive materials applicable in solar fuel research, electrosynthetic processes, photocatalytic environmental remediation, and photoelectrochemical sensing.

## Supplementary Material



## Notes

Details of the materials synthesis. Details of the photoBIA cell. Results regarding the application of the photoBIA system (PDF)
