# Supplementary material for: Photoassisted Batch Injection Analysis (photoBIA): Monitoring Photoelectrochemical Activity of Catalysts
Source: ACS Omega. 2025 Nov 10;10(46):56701–7. doi: 10.1021/acsomega.5c09379 (PMC12658704; doi:10.1021/acsomega.5c09379)
Supplement: Supplementary file 1 [file ao5c09379_si_001.pdf]

Photo-Assisted Batch Injection Analysis (photoBIA): Monitoring Photoelectrochemical Activity of Catalysts

Luiz Eduardo Gomes,[a] Vitória E. M. Icassatti,[a] Andréa L. C. D. de Avelar,[a] Zanib Qazi,[a] Rodrigo A. B. da Silva,[b] Heberton Wender,*[a] Cauê A. Martins*[a]

[a]Institute of Physics, Universidade Federal de Mato Grosso do Sul, CP 549, 79070-900, Campo Grande, MS, Brazil.

[b] Institute of Chemistry , Universidade Federal de Uberlândia (UFU), 38400-902, Uberlândia, MG, Brazil.

Corresponding Author: *heberton.wender@ufms.br, *caue.martins@ufms.br

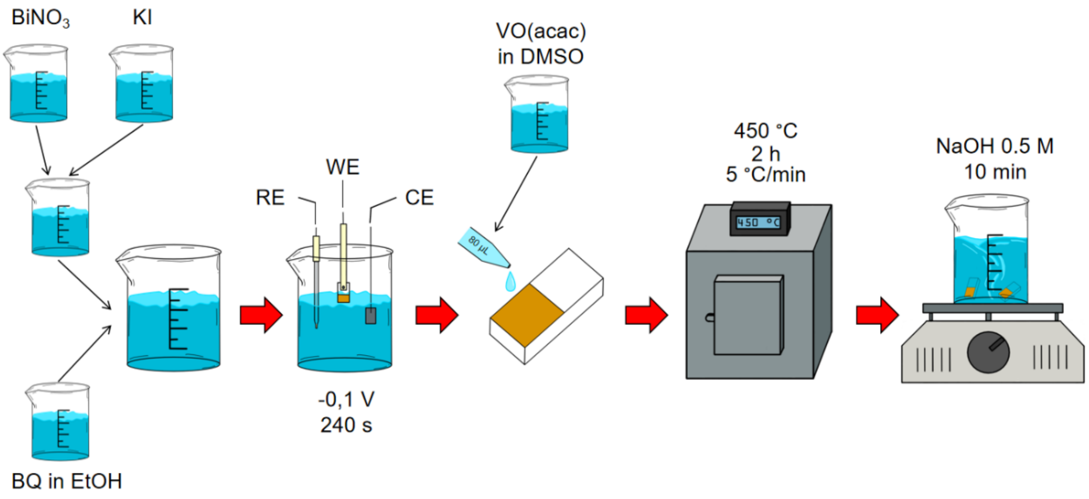

Figure S1. Schematic representation of the $BiVO_4$ photoanode preparation steps. First, a solution containing $Bi(NO_3)_3$, KI, and benzoquinone (BQ) in ethanol is prepared and used for the electrodeposition of BiOI onto an FTO substrate using a three-electrode electrochemical cell (applied potential = - 0.1 V vs. Ag/AgCl for 240 s). Then, the resulting film is drop-cast with a solution of $VO(acac)_2$ in DMSO. After drying, the electrode is annealed at 450 °C for 2 h with a heating rate of 5 °C/min. Finally, the material undergoes a post-treatment in 0.5 M NaOH solution for 10 minutes under stirring to remove excess of residual vanadium oxide phases, as previously described [1].

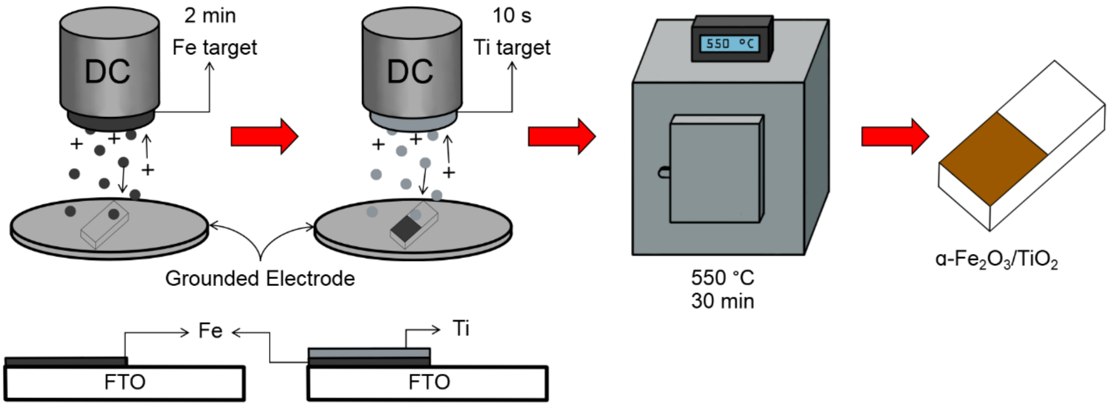

Figure S2. Schematic representation of the α-Fe_2O_3/TiO_2 preparation steps. First, an iron (Fe) thin film was deposited onto a fluorine-doped tin oxide (FTO) substrate by DC magnetron sputtering under an argon flow rate of 50 sccm and a sputtering power of 50 W for 2 minutes. Subsequently, a thin titanium (Ti) layer was deposited on top of the Fe film under identical sputtering conditions for 10 seconds. The resulting sample was then annealed at 550 °C for 30 minutes in air to promote crystallization Ti-modified hematite (α-Fe_2O_3/TiO_2) structure.

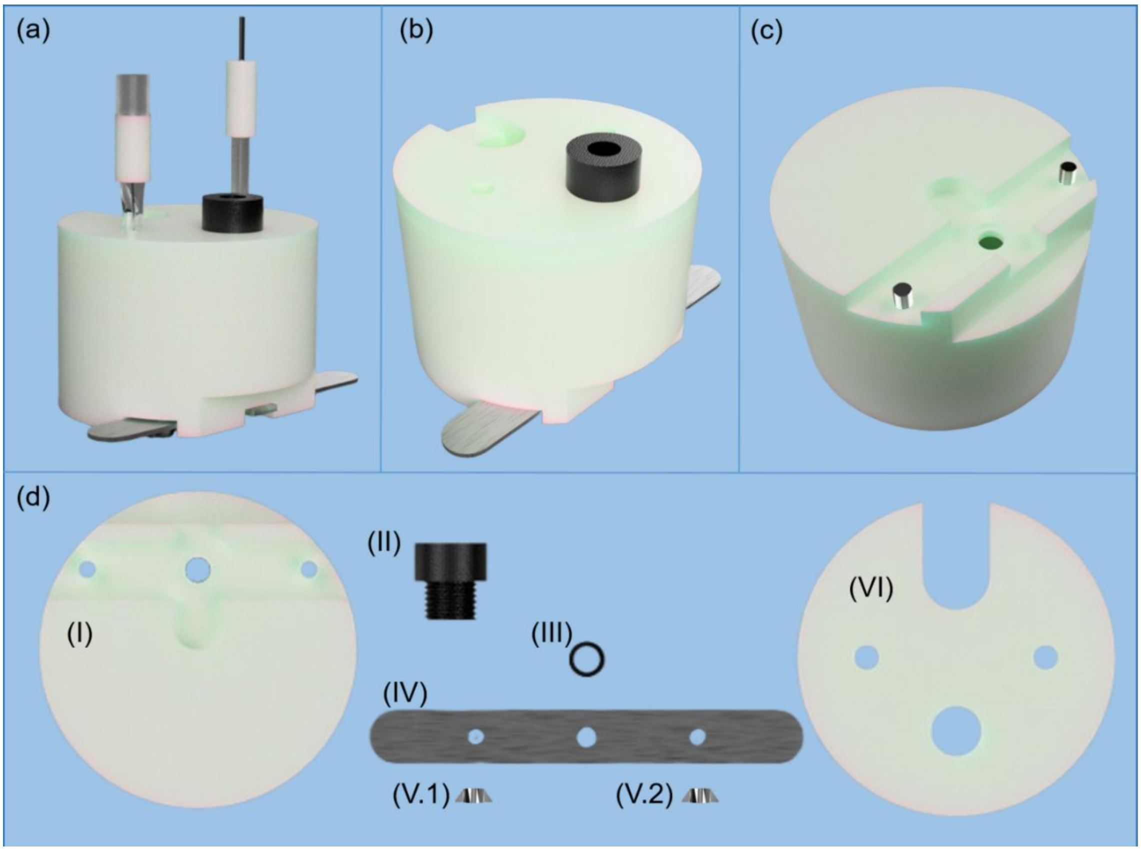

Figure S3. Design and components of the custom-built electrochemical cell used in the BIA system. (a) Fully assembled cell with the three-electrode configuration (WE, RE and CE). (b) Top view of the cell showing the sample injection port (black component). (c) Bottom view of the cell base, where the photoanode ($BiVO_4$-coated FTO) is positioned in the central orifice and illuminated from below during measurements. (d) Overview of all individual components of the cell, including the main body, bottom (I) and top (VI) covers, injection-port adapter (II), O-ring (III), electrode support plate (IV), and locking nuts (V.1 and V.2) [2,3].

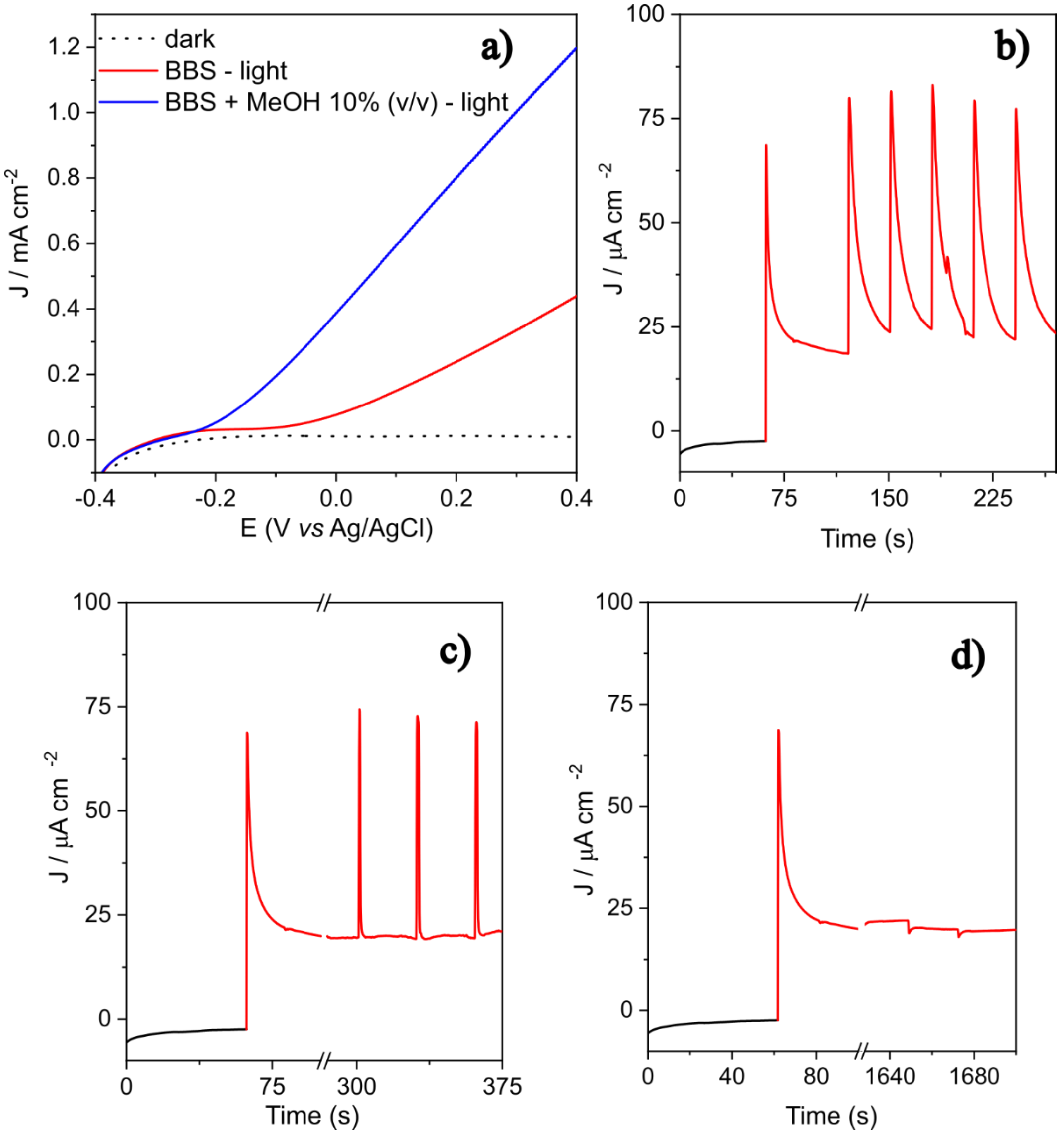

Figure S4. (a) Linear sweep voltammetry of $BiVO_4$ blank borate buffer solution (BBS) and 10% (v/v) methanol in BBS in a conventional cell under dark and light conditions. Chronoamperometric responses obtained in the photoBIA cell under continuous illumination: (b) sequential methanol injections without stirring, (c) sequential methanol injections with mechanical stirring, and (d) sequential injections of BBS only (without methanol) without stirring. Conditions for (b–d): injection volume of 100 µL; Eap = – 0.1 V.

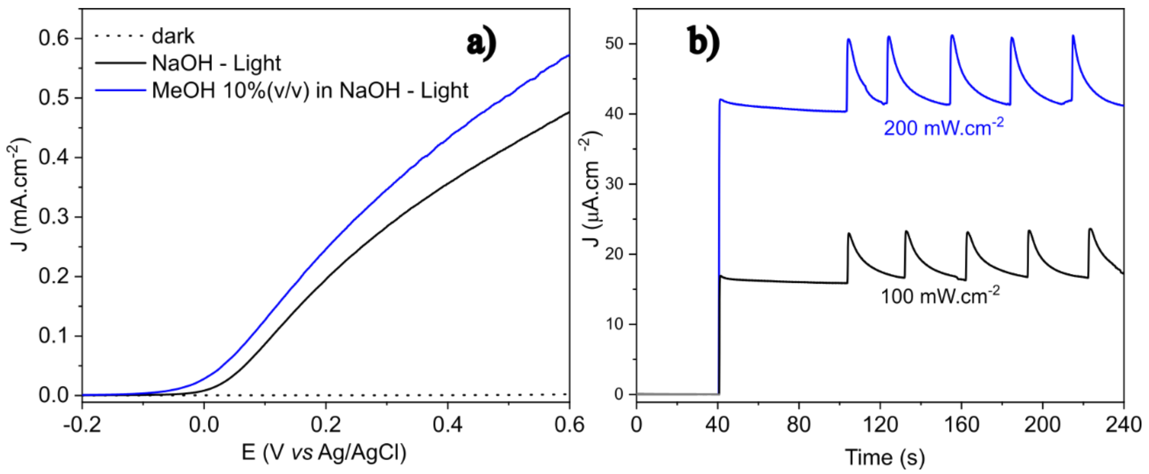

Figure S5. (a) Methanol photoelectrooxidation on α-Fe_2O_3/TiO_2 in a conventional stationary cell (Scan rate = 10 mV s^{-1}) in 1.0 mol L^{-1} NaOH under dark and illuminated conditions. (b) Transients of methanol photoelectrooxidation measured in a photoBIA system with amperometric detection (E = 0.4 V) under different illumination intensities of 100 mW cm^{-2} (black) and 200 mW cm^{-2} (blue).

References

[1] A.P. Rodrigues, M.J.M. Zapata, C.P. Ricardo, C.A. Martins, H. Wender, Cobalt-doping and CoOx cocatalyst synergistically enhances BiVO4 photoelectrochemical water and methane oxidation, Mater Lett 392 (2025) 138569. https://doi.org/10.1016/j.matlet.2025.138569.

[2] C. Garcia Cardozo, R. Melo Cardoso, T. Matheus Guimarães Selva, A. Evaristo de Carvalho, W. Torres Pio dos Santos, T. Regis Longo Cesar Paixão, R. Amorim Bezerra da Silva, Batch Injection Analysis-Multiple Pulse Amperometric Fingerprint: A Simple Approach for Fast On-site Screening of Drugs, Electroanalysis 29 (2017) 2847–2854. https://doi.org/10.1002/elan.201700520.

[3] P.F. Pereira, M.C. Marra, R.A.A. Munoz, E.M. Richter, Fast batch injection analysis system for on-site determination of ethanol in gasohol and fuel ethanol, Talanta 90 (2012) 99–102. https://doi.org/10.1016/j.talanta.2012.01.004.
